# Mechanisms of Generative Image-to-Image Translation Networks

**DOI:** 10.1109/access.2025.3638280

**Published:** 2025-11-27

**Authors:** GUANGZONG CHEN, MINGUI SUN, ZHI-HONG MAO, KANGNI LIU, WENYAN JIA

**Affiliations:** 1 Department of Electrical and Computer Engineering, University of Pittsburgh, Pittsburgh, PA 15260, USA; 2 Department of Neurosurgery, University of Pittsburgh, Pittsburgh, PA 15260, USA; 3 Department of Bioengineering, University of Pittsburgh, Pittsburgh, PA 15260, USA

**Keywords:** Adversarial training, autoencoders, generative adversarial networks (GANs), image-to-image translation, representation learning, simplified network architecture, style transfer, unsupervised translation

## Abstract

Existing image-to-image translation models often rely on complex architectures with multiple loss terms, making them difficult to interpret and computationally expensive. This paper is motivated by the need for a simpler, more fundamental understanding of the underlying mechanisms in image-to-image translations. We use a streamlined Generative Adversarial Network (GAN) that eliminates the need for auxiliary loss functions, such as cycle consistency or identity loss, which are common in state-of-the-art models. Our primary contribution is a theoretical and experimental demonstration that a basic GAN architecture is sufficient for high-quality image-to-image translation. We establish a connection between GANs and autoencoders, providing a clear rationale for how adversarial training alone can preserve content while transforming style. To validate our approach, we conduct experiments on several benchmark datasets and evaluate the performance of our simplified model, which achieves comparable results to more complex architectures. Our work demystifies the role of adversarial loss and offers a more efficient and interpretable framework for image-to-image translation.

## I. INTRODUCTION

The advancement of large neural networks has significantly improved the performance of image-to-image translation tasks. Its high accuracy and flexibility have attracted many researchers in various fields. Industries such as healthcare, automotive, and entertainment utilize image-to-image translation technologies for various applications, including medical imaging, autonomous driving, and digital content creation [1], [2], [3]. In addition, researchers in academia and the private sector are continuously innovating to explore new possibilities and advances in this area. Image-to-image translation encompasses a wide range of tasks, including edge-to-image, photo-to-painting, etc. [1], [4], and [5]. All of these tasks require significant computational and data resources for training models. Depending on the complexity of the model and the size of the dataset, training can take from hours to weeks.

A myriad of methodologies have been advanced to address the image-to-image translation problem. Although most existing models can solve the problem, they do not explain the mechanisms by which the network distinguishes content from style [6], [7], [8], [9], [10]. The nebulous definitions of content and style pose significant challenges in the mathematical characterization of the image translation process. Moreover, existing models for image-to-image translation often employ Generative Adversarial Networks (GANs) architecture, but encompass significant complexity, incorporating elements such as cycle loss, identity loss, and penalties on intermediate features. The necessity of these intricate penalties is rarely examined.

Previously, we introduced a GAN-based model to transform food images using only a GAN penalty without any additional penalties [4]. In this paper, we investigate the similarity between Generative Adversarial Networks (GANs) [11] and autoencoders [12] to elucidate the GAN model mechanism for image-to-image translation without imposing additional penalties. Subsequently, we show the rationale behind the efficacy of employing only the GAN component for image-to-image translation tasks. We offer a clear explanation that substantiates the primary role of GAN components in addressing the image-to-image translation problem.

We have conducted a comprehensive review and analysis of the models employed for image generation and image-to-image translation. Our investigation focuses on identifying the efficacy of various components of the network. Notably, we discovered that the autoencoder and GAN models generate homologous output and provide an explanation for this phenomenon. This explanation also extends to the efficiency of GANs in the context of image-to-image translation. From our perspective, we employ a preliminary GAN for image-to-image translation. Furthermore, our findings elucidate why some examples in the network may fail.

The main contributions of this paper are highlighted as follows: (i) We offer a comprehensive explanation of using GANs in the context of image-to-image translation, demonstrating that a simple form of GANs can accomplish translation tasks as well as the GANs with more complex structures. (ii) We demonstrate that, with a discriminator capable of distinguishing between real and synthetic images, adversarial training for autoencoder models yields results similar to those of traditional autoencoder models. This is substantiated through experimental validation. (iii) We extend adversarial training to address the image-to-image translation problem, illustrating that a straightforward GAN model can preserve common features and generate novel ones, whereas previous methods impose additional penalties to maintain common features. (iv) Our work provides a rationale for the efficacy of GANs in the image-to-image translation context, clarifying that the decomposition of texture and content signifies common and differentiating characteristics determined by the dataset. This offers a more precise and comprehensive understanding compared to previous studies.

The paper is structured as follows: The Related Works section gives a brief review of image generation and translation. The Methods section provides our explanation, encompassing algebraic and geometric interpretations. Subsequently, the experiment section presents three experiments. The first experiment compares the performance of GANs and autoencoders, the second investigates the model’s capability for image-to-image translation, and the third examines the constraints outlined in the Methods section. Finally, conclusions are drawn based on our analysis.

## II. RELATED WORKS

### A. GENERATIVE ADVERSARIAL NETWORKS (GANS)

GANs are widely utilized for image generation. These architectures are composed of a generator (G) and a discriminator (D) that compete in a min-max game during training. Numerous variations of GANs have been proposed to enhance their performance, such as CGAN [13], [14], [15], CVAE-GAN [16], VQ-GAN [17], StyleGAN [18], GigaGAN [19] and so on [20]. Additionally, extensive research has been conducted to address issues such as mode collapse and unstable training [20]. These contributions substantially advance the capability of GANs in producing high-fidelity images.

### B. IMAGE TRANSLATION

Gatys et al. proposed a seminal approach in which they demonstrated that style and content could be separated within a convolutional network. They used feature maps to capture the content and a Gram matrix to capture the style [21]. Style transfer has become increasingly popular with many researchers. Furthermore, numerous models have been introduced for image-to-image translation. CycleGAN [6], DualGAN [7], and similar models posited that the transformation between two domains should be invertible. These models used two GANs to learn invertible image translation. Other approaches, like MUNIT [8], DRIT++ [22], and TransferI2I [9], assumed that style and content are controlled by different sets of latent variables. Based on this assumption, they developed various network structures to achieve the desired translations. Palette employs a diffusion model for image-to-image translation [5]. However, its applicability is limited to tasks such as inpainting, colorization, and uncropping.

Zheng et al. [23] addressed the issue of imbalanced image datasets using a multi-adversarial framework. In addition, they introduce an asynchronous generative adversarial network to boost model performance. Yang et al. enhance the quality of the generated images through semantic cooperative shape perception [24]. Additionally, researchers apply various techniques such as multi-constraints, semantic integration, and a unified circular framework to refine image-to-image translation models by modifying model specifics [25], [26], [27], [28], [29], [30].

### C. NETWORK EXPLANATION

Besides these models that provide methods for image-to-image translation, a variety of approaches have been suggested to clarify the fundamental processes driving the network’s functioning from different analytical perspectives.

Classification models are essential elements of GANs. The foundational theory underlying these models is vital for the proper function of GANs. Yarotsky established error limits for networks [31], while Wang and Ma determined error bounds for both multi-layer perceptrons and convolutional neural networks. These studies demonstrate the theoretical correctness of convolutional neural networks [32].

Beyond the classification model, Ye et al. introduced deep convolutional framelets as described in [33]. They utilized deep convolutional framelets to explain a model comparable to U-Net, proposing an approach that captures finer details than U-Net. This model helps to comprehend the roles of various components, such as the number of features, skip connections, and concatenation within the network.

In the context of generator networks, the variational autoencoder (VAE) and diffusion models are well explained [12], [34], [35]. The VAE focuses on minimizing the evidence lower bound (ELBO), whereas the diffusion model views the network’s process as a Markov chain and derives its loss function based on the characteristics of a Markov chain. Generally, a GAN model trains a model that distinguishes the difference between real and fake. However, when GANs are applied to image-to-image translation tasks, a significant portion of the research centers on developing heuristic models, and much of the interpretation of these models is heuristic.

## III. METHODS

The aim of this section is to elucidate the mechanism of adversarial training within the context of image-to-image translation challenges. Initially, we focus on a specific instance: the identity image translation task. Subsequently, we broaden our analysis to encompass the general image-to-image translation paradigm, providing a comprehensive explanation to demonstrate how GAN models can be applied to image-to-image translation tasks.

The task of recovering an image from a latent space is commonly addressed through autoencoders. This issue is similar to image reconstruction. However, in image reconstruction, the input image may exhibit certain defects that require correction. In contrast, in our scenario, the input and output images are identical. Our findings demonstrate that employing either of the two methodologies yields similar results. Consequently, these conclusions can be extrapolated to the image translation problem.

Autoencoders are widely employed to derive latent variables from input images. It is also used in image reconstruction applications. The main objective of an autoencoder is to learn a mapping function G(x), capable of reconstructing the input image x. The generator G, comprises an encoder and a decoder, where the encoder is utilized to obtain the latent variable and the decoder reconstructs the image from the latent variable.

Adversarial training, in this paper, is defined by the introduction of a mapping function D which makes apparent the differences between authentic images x and reconstructed images G(x). It is similar to the discriminator function in a GAN. The difference between the mapping function D and the discriminator in a GAN is that D does not use a binary output while the discriminator function in a GAN requires a binary output. The discriminator in GANs is a special case of the mapping function D. The training framework is a min-max game between G and D, in which D aims to maximize the loss function, while G aims to minimize it.

**FIGURE 1.**
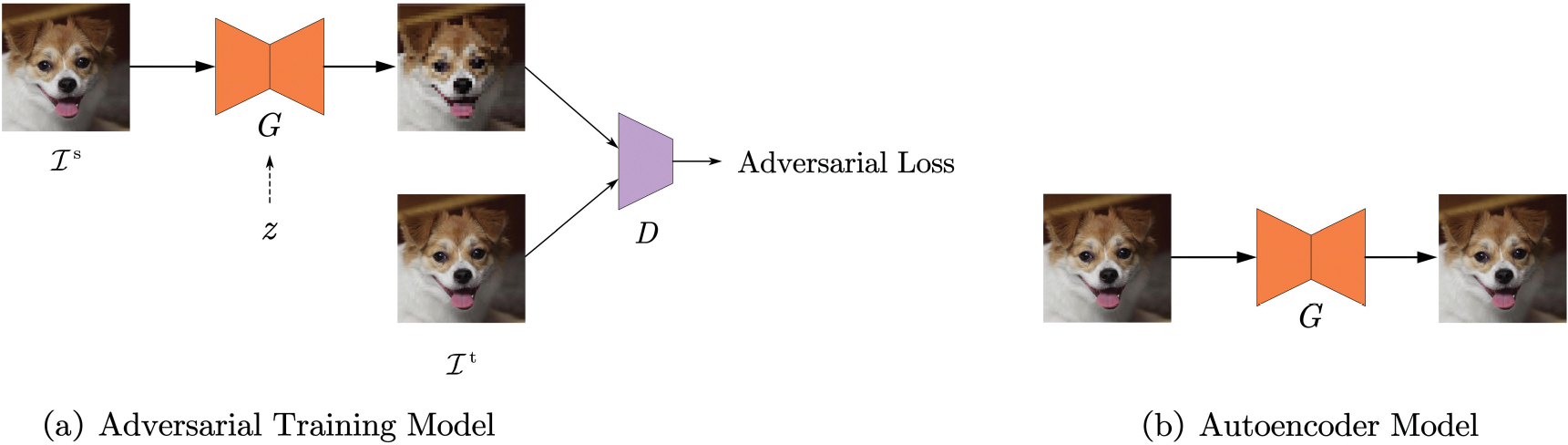
Architecture of the Method. Left: The adversarial training model includes a generator (G) to produce images and a discriminator (D) to distinguish generated images. Datasets ${ℐ}^{s}$ and ${ℐ}^{t}$ provide shape and texture information. Variable z introduces variability. Right: The autoencoder model is used to reconstruct the input image. This paper shows that these two models converge to the same results under certain conditions.

Fig. 1 shows two distinct network architectures for generative learning and autoencoder. The right is adversarial training. The left is the autoencoder. For the autoencoder, the goal is to employ a model to recreate the input data. Adversarial training involves alternating the learning of G and D, where G generates images and D identifies the differences between the input and the generated output. A random variable z is sampled from a Gaussian distribution and used exclusively to produce multiple outputs from a single input image. The image datasets ${ℐ}^{s}$ and ${ℐ}^{t}$ represent distinct datasets, where ${ℐ}^{s}$ is used as shape references and ${ℐ}^{t}$ is used to provide texture information. When comparing the autoencoder with adversarial training, we set ${ℐ}^{s}$ and ${ℐ}^{t}$ to be identical.

### A. SIMILARITY BETWEEN AUTOENCODER AND ADVERSARIAL TRAINING UNDER CERTAIN CONDITION

In this subsection, we demonstrate that autoencoders and adversarial training yield similar results given two specific constraints. Firstly, the generator must have the ability to reconstruct the input image. Secondly, the mapping function D should accurately perceive the distinction between x and G(x).

#### 1) ALGEBRAIC EXPLANATION

Let $ℐ=\left\{x^{\left(1\right)},x^{\left(2\right)},\ldots ,x^{\left(m\right)}\right\}$ be a set of data, where $x^{\left(i\right)}={\left[x_{1}^{\left(i\right)},x_{2}^{\left(i\right)},\ldots ,x_{n}^{\left(i\right)}\right]}^{T}\in R^{n}$.

The optimization problem of the autoencoder is formulated as:

(1) $$\underset{G}{min}\;L=\frac{1}{m}\sum_{x\in ℐ}\|x-G(x)\|$$

where ‖⋅‖ is the $L_{1}$ norm, which is the sum of the absolute values of the elements of a vector.

The adversarial training incorporates an additional mapping function D, which maps x to a vector D(x), with D(x) belonging to $R^{n}$. After transformation, in the new space, D(x) and D(G(x)) are linearly separable. It is important to note that the GAN requires a binary output from the discriminator, whereas the mapping function D projects to a new space with dimension n.

The optimization problem of adversarial training is defined as follows:

(2) $$\underset{G}{min}\;\underset{D}{max}\;L=\frac{1}{m}\sum_{x\in ℐ}\|D(x)-D(G(x))\|$$

where D(x) equals ${\left[D_{1}(x),D_{2}(x),\cdots ,D_{n}(x)\right]}^{T}$.

The main difference between autoencoders and adversarial training is the presence of an auxiliary function D. This additional component augments the differences between the input data points x and their generated data G(x), which helps to train the generator. Both algorithms aim to make G(x) close to x, leading to similar results. However, they might produce different results because, near the optimal solution, D in adversarial training can become oscillating, causing G to fluctuate around the optimum. In contrast, the autoencoder will converge to the optimal solution.

In (2), the training data is paired, which means x and G(x) must be considered together when computing the loss function. We will now demonstrate that adversarial training can be performed without paired data. If the function D can maximize the loss function and perfectly distinguish between x and G(X) on each feature, then there must be a function D such that $D_{i}(x)>D_{i}(G(x))$ and optimize the loss function at the same time. Then we have the following loss function:

(3) $$L=\frac{1}{m}\sum_{x\in ℐ}\sum_{i}\left[D_{i}(x)-D_{i}(G(x))\right].$$

Rearranging the equation, we have:

(4) $$L=\frac{1}{m}\left[\sum_{x\in ℐ}\;\sum_{i}\;D_{i}(x)-\sum_{x\in ℐ}\;\sum_{i}\;D_{i}(G(x))\right].$$

We can define another function $\overset{ˆ}{D}(x)=\sum_{i}D_{i}(x)$, where $\overset{ˆ}{D}(x)\in R$. And the loss function can be written as:

(5) $$L=\frac{1}{m}\left[\sum_{x\in ℐ}\;\overset{ˆ}{D}(x)-\sum_{x\in ℐ}\;\overset{ˆ}{D}(G(x))\right].$$

Because D is only required to distinguish different features in x and G(x), we consider using random variables and distribution to model the problem. Let $p_{\text{data}}$ be the distribution of the data set and $p_{\text{g}}$ be the distribution of the generator’s output, and replacing the average with the expectation, then we have

(6) $$L=E_{x∼p_{\text{data}}(x)}[\overset{ˆ}{D}(x)]-E_{x∼p_{g}(x)}[\overset{ˆ}{D}(x)].$$

This is similar to the WGAN loss function [36]. From (2), we know that G(x) will be pushed to x when minimizing the loss function. Therefore, adversarial training should produce results similar to autoencoder models. Equation (6) tells us that if the discriminator D can perfectly distinguish the data from $p_{\text{data}}$ and $p_{g}$, the loss function will not depend on the order of x and G(x).

#### 2) GEOMETRIC INTERPRETATION

**FIGURE 2.**
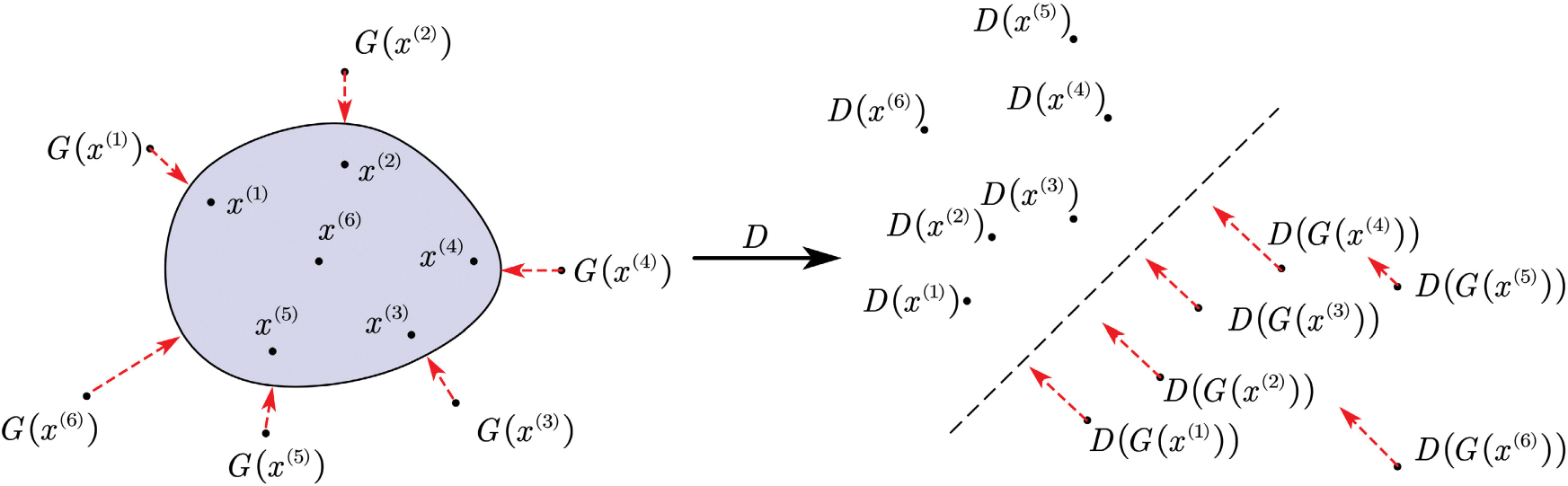
Geometric representation of initial phase of the model.

We also present a geometric interpretation of why adversarial training can produce results similar to the autoencoder. Fig. 2 shows the status of the early stage of the model training. After learning D(·) in the max part of the min-max optimization problem (2), we project the values of x and G(x) onto a new feature space where the set of x projections and the set of G(x) projections are well clustered and can be separated by a hyperplane—a linear boundary, similar to how the data with different labels are separated in the support vector machine (SVM). If we map the dividing surface to the original space, a nonlinear boundary will emerge to distinguish x values from G(x) values. When solving the min part of the min-max optimization problem for G(·), the G(x) values will move toward the boundary, getting closer to the x values, as demonstrated by the red arrows in Fig. 2. Through the alternating training of G and D, the set of G(x) becomes closer to the set of x, effectively pushing both G(x) and x values toward the boundary. This process is likely to bring each pair of G(x) and x close to each other.

**FIGURE 3.**
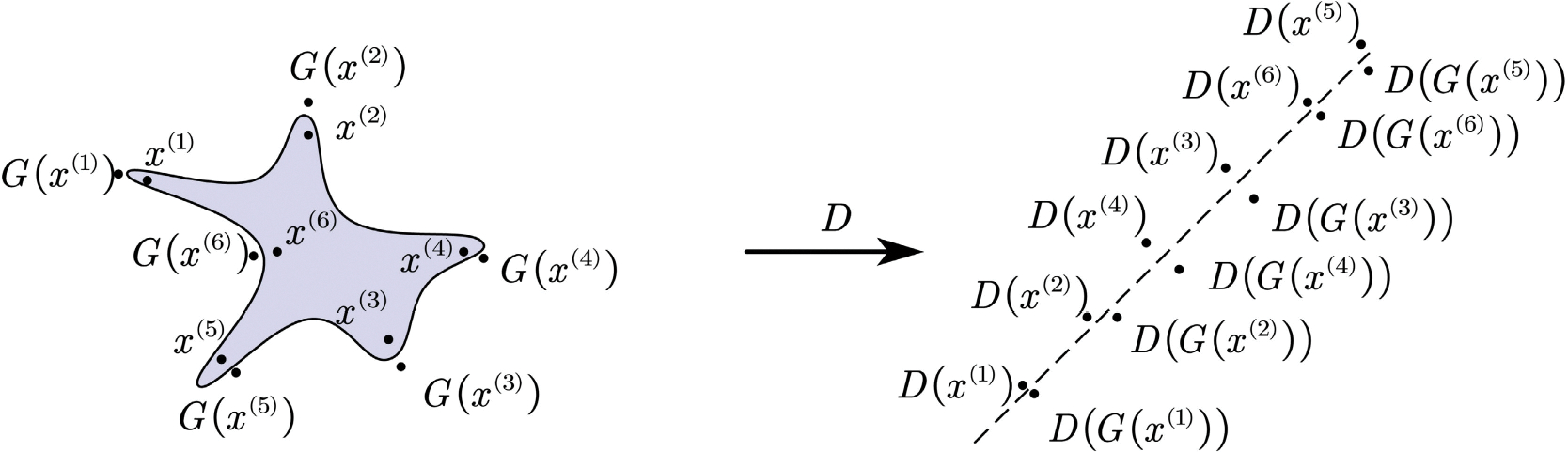
Geometric representation of the model after alternating training G and D.

Fig. 3 illustrates the effects of G(·) and D(·) after training. Within the transformed space, D(x) and D(G(x)) values are distributed along the hyperplane. In the original space, the boundary is nonlinear, and x and G(x) values scatter close to each other.

From this perspective, the result of adversarial training will be similar to that of the autoencoder when ${ℐ}^{s}$ and ${ℐ}^{t}$ are from the same distribution. This observation may contradict our initial expectations that GANs could generate any sample that fits the distribution of the dataset. However, our findings indicate that the adversarial model will produce the input data without imposing a reconstruction penalty between x and G(x).

### B. IMAGE-TO-IMAGE TRANSLATION

The network architecture is depicted on the left of Fig. 1. It incorporates two datasets: The first image dataset, ${ℐ}^{s}$, is used as a shape reference, where ${ℐ}^{s}$ equals $\left\{I_{i}^{s}|i=\right.1,\ldots ,N\},I_{i}^{s}\in R^{H\times W\times 3},H$ and W are the height and width of the images, 3 is the number of channels of an RGB image, and N is the total number of images. The second image dataset, ${ℐ}^{t}$, is used to provide texture information, where ${ℐ}^{t}$ equals $\left\{I_{i}^{t}|i=1,\ldots ,M\right\},I_{i}^{t}\in R^{H\times W\times 3}$, and M is the size of the second dataset. This dataset is provided to the discriminator D, to train the network.

We want to apply ${ℐ}^{s}$ to facilitate the network to generate images with the same shapes as the images in ${ℐ}^{s}$ while maintaining the textures from ${ℐ}^{t}$. For example, zebras and horses share a common body shape but differ in texture. The dataset ${ℐ}^{s}$ comprises horse images, whereas ${ℐ}^{t}$ consists of zebra images. Image translations aim to substitute the horse image texture with that of the zebra.

In the self-translation task, the mapping function D is required to verify that all features in both x and G(x) are identical. On the other hand, in the image-to-image translation task, the discriminator’s role is to confirm that all features in the generated image match the distribution of ${ℐ}^{\text{t}}$.

Consider an input image with two feature sets, $x=\left[x_{1},x_{2}\right]$, where $x_{1}$ appears in both ${ℐ}^{s}$ and ${ℐ}^{t}$, but $x_{2}$ is only found in ${ℐ}^{t}$. In this case, the network will preserve the feature $x_{1}$ and substitute $x_{2}$ with a feature from the ${ℐ}^{t}$ dataset. The preservation of $x_{1}$ was explained in the previous section. Adversarial training will maintain all features if they are present in the ${ℐ}^{t}$ dataset.

## IV. EXPERIMENTAL RESULTS AND DISCUSSIONS

We conducted three experiments. First, we verified our theoretical finding that GANs produce results similar to those of autoencoder models when the reference images ${ℐ}^{s}$ and texture images ${ℐ}^{t}$ are the same. Second, we showcased the GAN model’s capability to transform images from one domain to another. Lastly, we modified the dataset size and the generator configuration to examine the impact of the constraints as discussed in the Methods section.

We evaluated our model on various datasets, such as Animal FacesHQ (AFHQ) [37], Photo-to-Van Gogh, Photo-to-Monet from CycleGAN [6], and Flickr-Faces-HQ (FFHQ) [18]. The AFHQ dataset consists of 16 130 images of animal faces, each with a 1 024 × 1 024 pixel resolution, covering three categories of animals: cat, dog, and wild. The Photo-to-Van Gogh and Photo-to-Monet have approximately 1000 images for each category. The FFHQ dataset is a high-quality collection of human facial images. It comprises 70 000 images, all at a resolution of 1 024 × 1 024 pixels. In this study, we resized images to 512 × 512 resolution for all experiments.

For both the generator and discriminator, we utilized StyleGAN v2 [18] as the foundational architecture. Given that an additional encoder is required to encode the image into features, we used a simple convolutional network as the encoder, which comprises only convolution, downsampling, and ReLU activation.

### A. COMPARISON BETWEEN GAN AND AUTOENCODER

In this subsection, we used the AFHQ dataset to demonstrate the correctness of our analysis in the Methods section.

**FIGURE 4.**
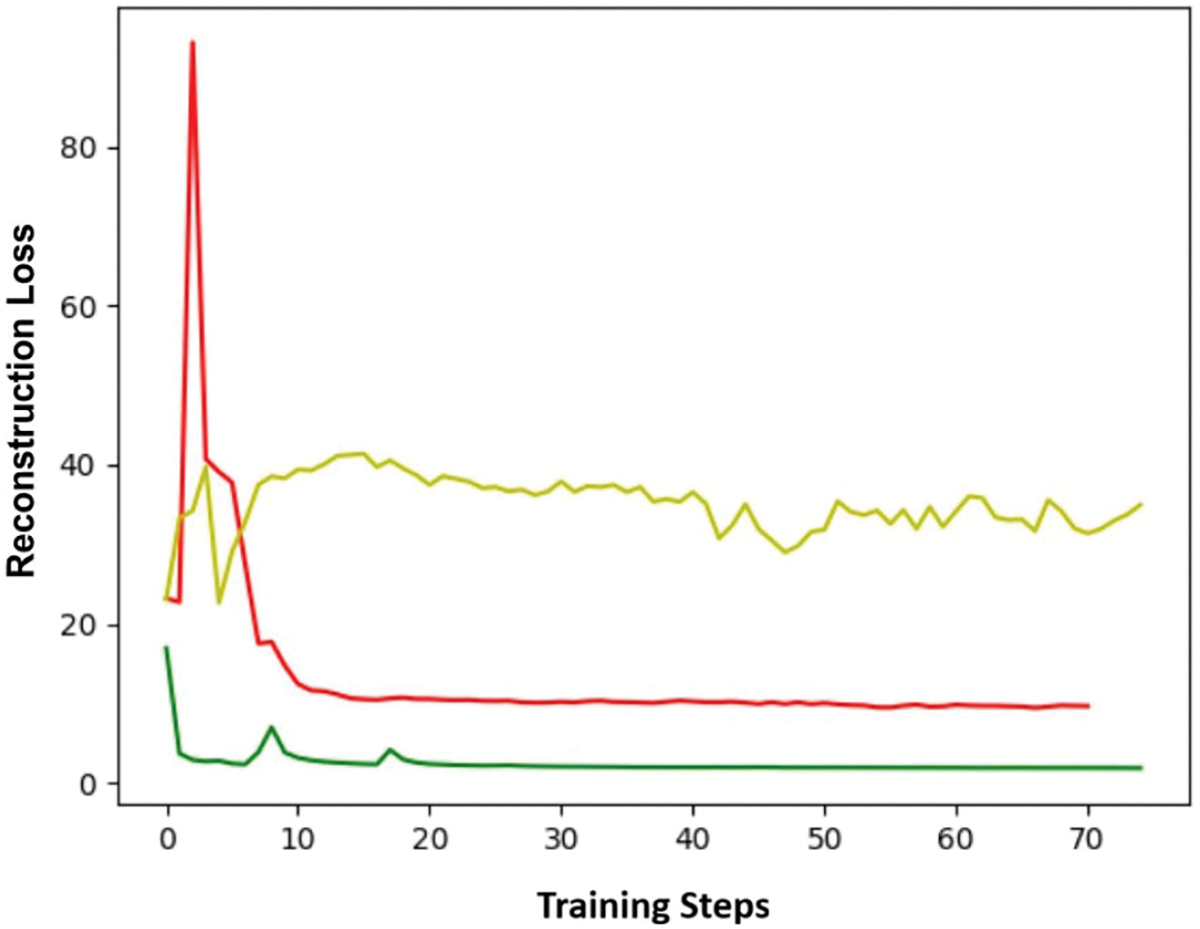
Reconstruction losses from three distinct training sessions. Green: Autoencoder; Red: GAN; Yellow: GAN for image-to-image translation.

We claim that GANs and autoencoders can produce similar results when the generator and discriminator have enough capacity. We used the mean square error between the original image and the generated image to evaluate the performance of the two models. Fig. 4 shows the reconstruction loss for three different models during the training phase.

To ensure a fair comparison between the GAN and the autoencoder, we computed the reconstruction loss for generations after every 1 000 images used to train the model. The green curve is generated by the autoencoder, the red curve by the GAN, and the yellow curve represents the reconstruction loss of the GAN model, when ${ℐ}^{s}$ and ${ℐ}^{t}$ differ. These findings suggest that when ${ℐ}^{s}$ and ${ℐ}^{t}$ are equivalent, both the GAN and autoencoder are effective in minimizing the reconstruction loss. Despite the fact that reconstruction loss is not utilized during the training of the GAN model, this reinforces the validity of our analysis in the Methods section.

**FIGURE 5.**
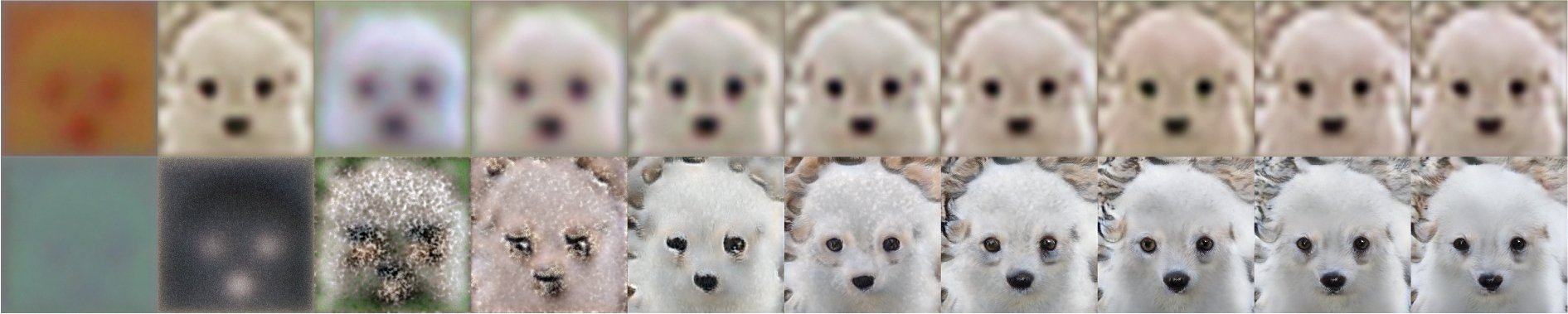
Intermediate results from the autoencoder and GAN, with the top row from the autoencoder, and the bottom row from the GAN.

Fig. 5 shows the outputs from the generator. This illustration makes it clear that the discriminator network starts by focusing on global features and then transitions to focusing on local features (first row of Fig. 5). In contrast, the autoencoder behaves differently, as it directly minimizes the loss across the entire image (second row of Fig. 5).

**FIGURE 6.**
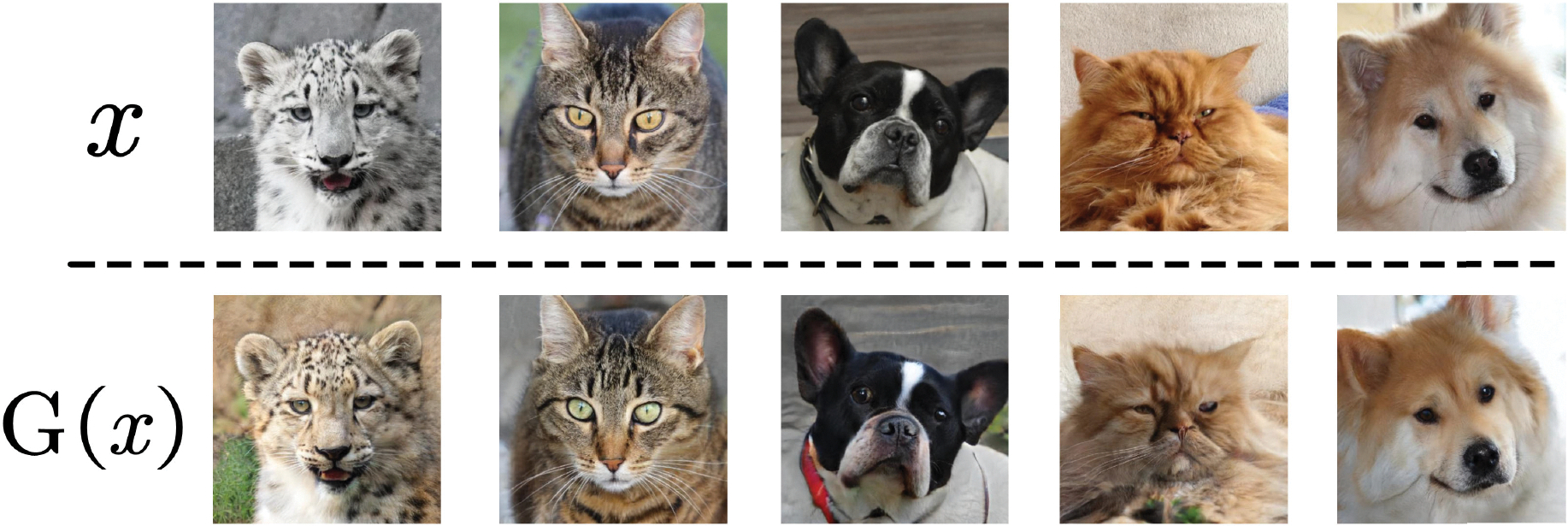
Input and generated images. The top row displays the original images, while the bottom row shows the generated images.

Fig. 6 shows both the original images and the generator’s outputs. This result indicates that the outputs of the generator are similar to the input images. However, there are noticeable differences between the input and output images, such as variations in color and background. This result also illustrates the gap between the GAN and autoencoder in Fig. 4. The GAN is capable of bringing G(x) close to x, but it cannot make them identical without incorporating a reconstruction loss.

### B. IMAGE-TO-IMAGE TRANSLATION CAPABILITY

When ${ℐ}^{s}$ and ${ℐ}^{t}$ are different, our method can be used for image-to-image translation and the same feature in both datasets will be preserved. Compared to other methods, the network is simpler, and we can predict the outcomes and provide explanations for the results.

**FIGURE 7.**
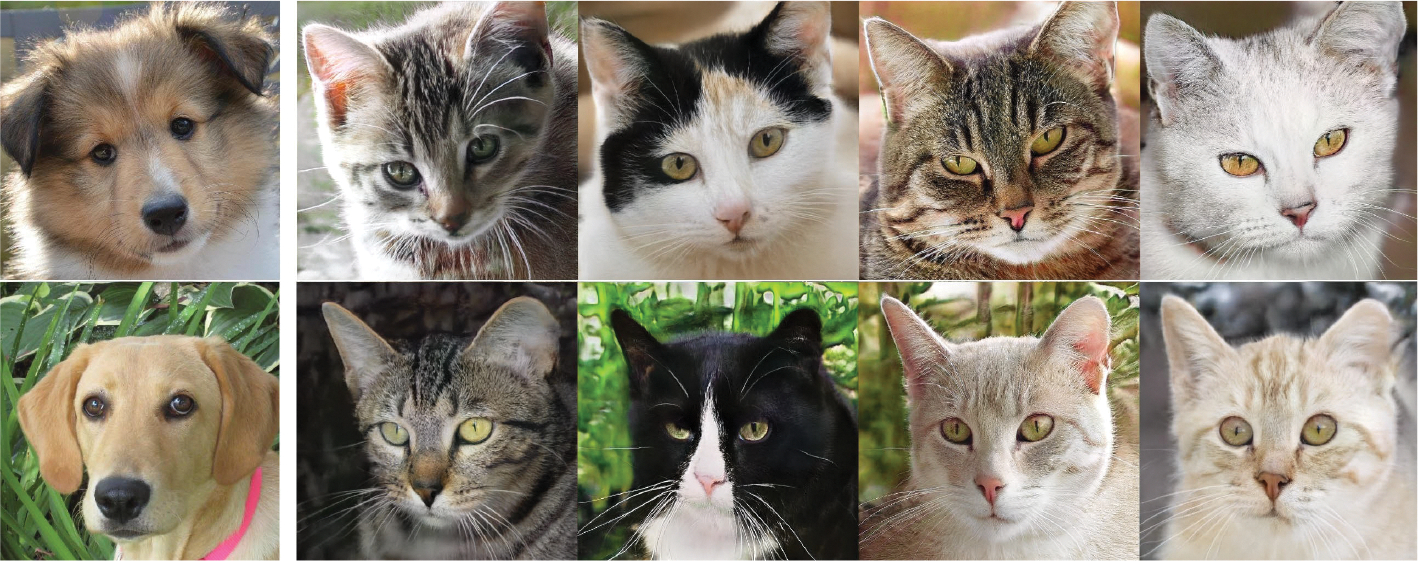
Results of animal image translation. First column is the input images and the rest are generated images.

Fig. 7 shows animal transfer examples. The first column displays the input, followed by four columns showing the outputs. The dogs’ faces are used as ${ℐ}^{s}$ and cats’ faces as ${ℐ}^{t}$. The generated cat face retains the same orientation as the dog’s face. In addition, the relative positions of facial features such as the eyes, nose, and ears remain uniform.

**FIGURE 8.**
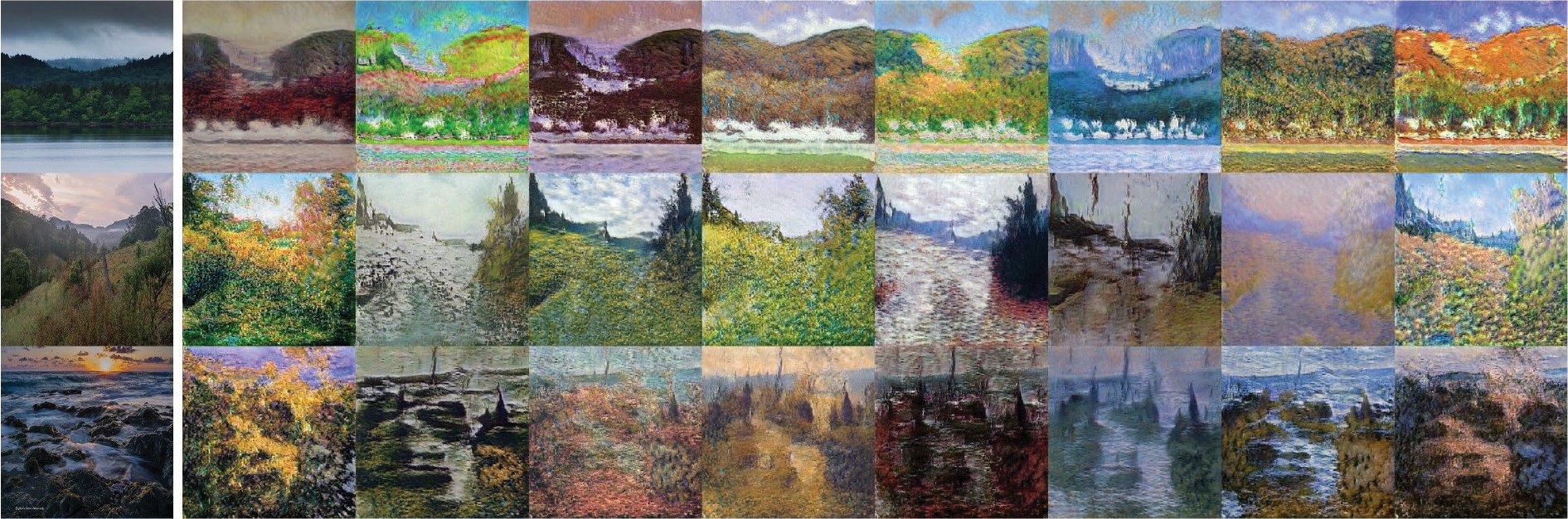
Translation from photo to Monet style. First column is the input image, and the rest are generated images.

A translation between an artwork and a photograph is also illustrated. In Fig. 8, the first column shows the input, while the subsequent columns show the output. It is noticeable that the objects remain the same, but the textures are different in the output. However, in the first row, the shape of the mountain appears slightly altered. According to our explanation, this happens because the input shape of the mountain is absent in the target dataset, causing the network to modify the mountain’s shape.

In both animal and artwork translations, it shows successes in preserving global topological characteristics. The results of these experiments show that our network can have similar results to other style transfer networks.

From this experiment, we can roughly tell what the shape (content) and style are in other style transfer models, while the other models did not explicitly indicate the content and style. In the AFHQ dataset, the style may refer to the breed of the animal, and the content refers to the pose and angle of the animal. In the Photo-to-Van Gogh dataset, the style refers to the color and texture of the picture, and the content refers to the objects in the picture. However, in our work, we can tell that the network does not have a semantic understanding of the image. The content actually refers to the common features in both datasets, and the style refers to the features only present in ${ℐ}^{t}$ but not in ${ℐ}^{s}$.

### C. CONSTRAINTS ANALYSIS

In the previous subsection, we demonstrated that our method can generate results similar to those of an autoencoder and also showed that the network has the capacity to solve image-to-image translation tasks. However, our method hinges on two critical conditions: first, the generator must be capable of completely reconstructing the input image; second, the discriminator must be able to perfectly distinguish between real and fake images whenever there is a discrepancy. In this subsection, we discuss the impact of these two conditions. We consider the generator to be composed of two parts: an encoder and a decoder. If the encoder’s capacity is insufficient, it can only retain certain features, implying that the generator will fail to produce an exact match of the input when dealing with a large dataset. Evaluating the condition on the discriminator is inherently challenging, but it is known that a smaller dataset makes it easier for the network to memorize the entire dataset. Therefore, we present results based on various dataset sizes.

We conducted two experiments to illustrate how the abovementioned two conditions influence the network’s performance. We employed both the FFHQ and AFHQ datasets, as they allow us to compare the effects of dataset size. We utilized varying sizes of intermediate features. Employing smaller intermediate features results in increased difficulty in reconstructing the input image. We find that the network initially captures the global topological features, followed by the detailed ones, which is the same as we observed in the first experiment. If the size of the dataset is sufficiently small, which means that the network has the ability to distinguish between *G*(*x*) and *x*, the network tends to converge towards a one-to-one mapping.

The first experiment kept the same encoder structure as in the previous experiment, where the intermediate feature is 16 × 16 × 128 referring as *high dimension feature*. In the second experiment, we added one more convolutional block in the encoder. The intermediate feature becomes 8 × 8 × 128 which is referred to as *low dimension features*. In low dimension features, the encoder makes the information more compact, and more information is lost.

#### 1) IMAGE TRANSLATION WITH HIGH DIMENSION FEATURES

**FIGURE 9.**
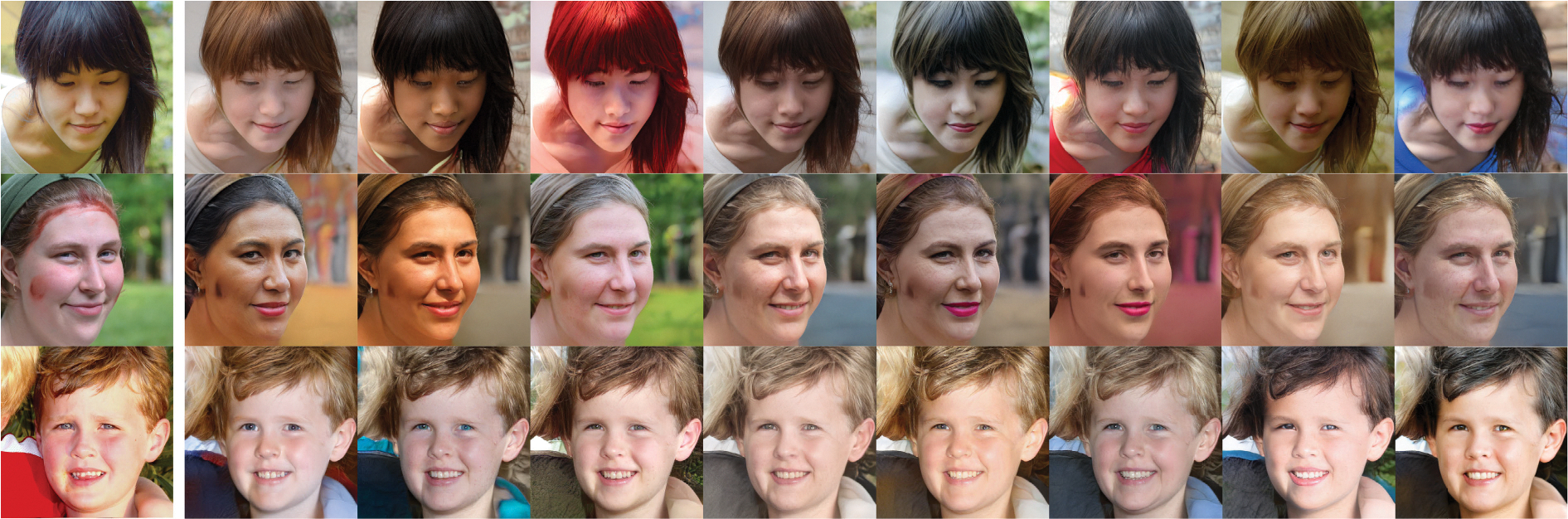
Face-to-face translation results with 16 × 16 × 128 intermediate features. The first column shows the input images, and the rest are the generated images.

The results of human face transfer are shown in Fig. 9. The first column is the input image and the following columns are the corresponding output images. The output images look like a series of selfies of similar people with different detailed textures. The global topology information, such as the positions of the eyes, nose, and mouth, is maintained in the same positions as the input. The detailed features, such as skin folds and hair color, are randomly set.

**FIGURE 10.**
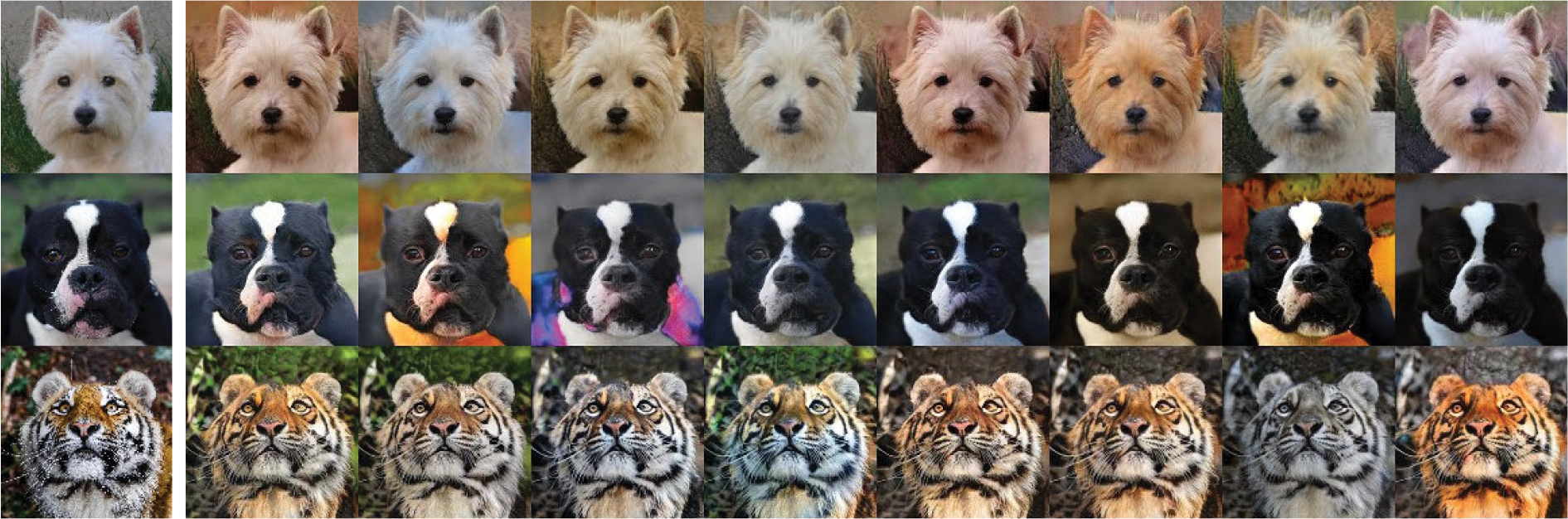
Animal-to-animal translation results with 16 × 16 × 128 intermediate features.

The same model was applied to the AFHQ dataset. However, the number of images is only 3 000, while the human face dataset has 70 000 images. The result is shown in Fig. 10. Compared to Fig. 9, the only difference is the colors of the output images in the same row. All other features remain the same.

The varying outcomes of the two experiments are due to differences in the sizes of the datasets. When the size of the dataset is relatively low, the discriminator possesses sufficient capability to distinguish differences, causing the output of the GAN to converge to that of the autoencoder. This demonstrates the validity of the analysis in the Methods section.

#### 2) IMAGE TRANSLATION WITH LOW DIMENSION FEATURES TRANSFER

**FIGURE 11.**
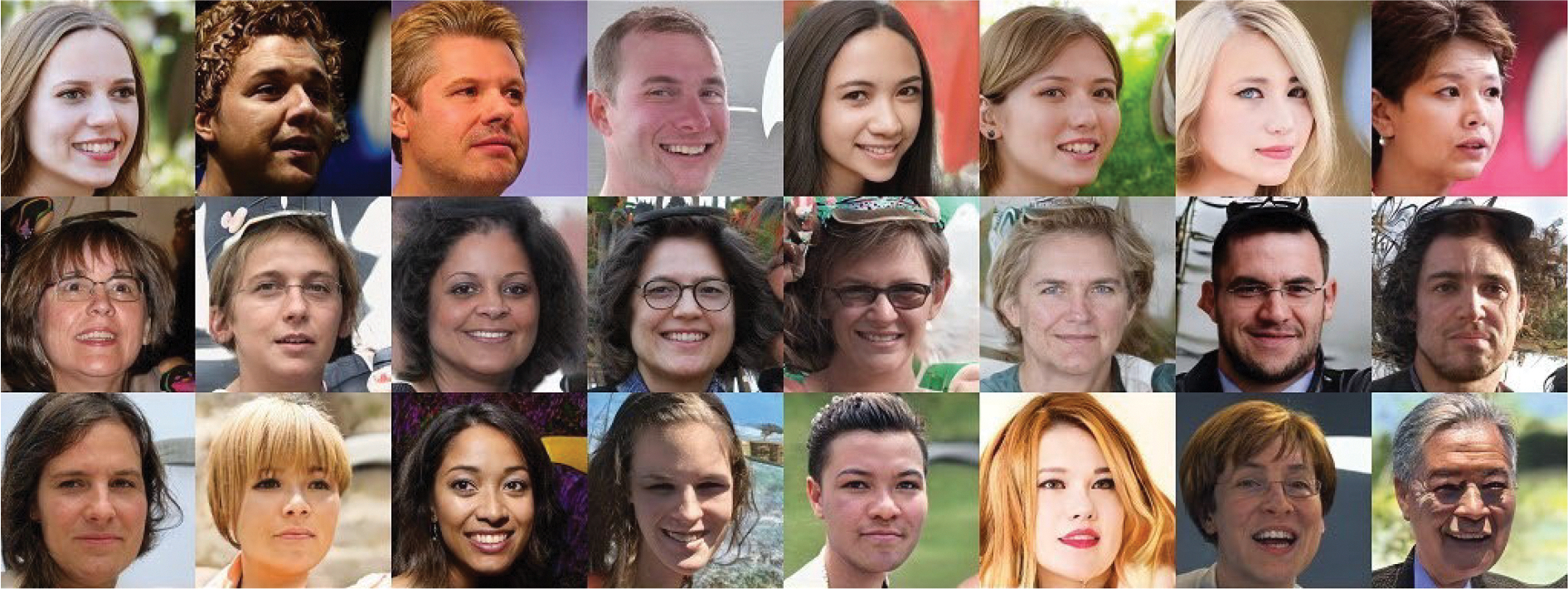
Face-to-face translation result with 8 × 8 × 128 intermediate features.

**FIGURE 12.**
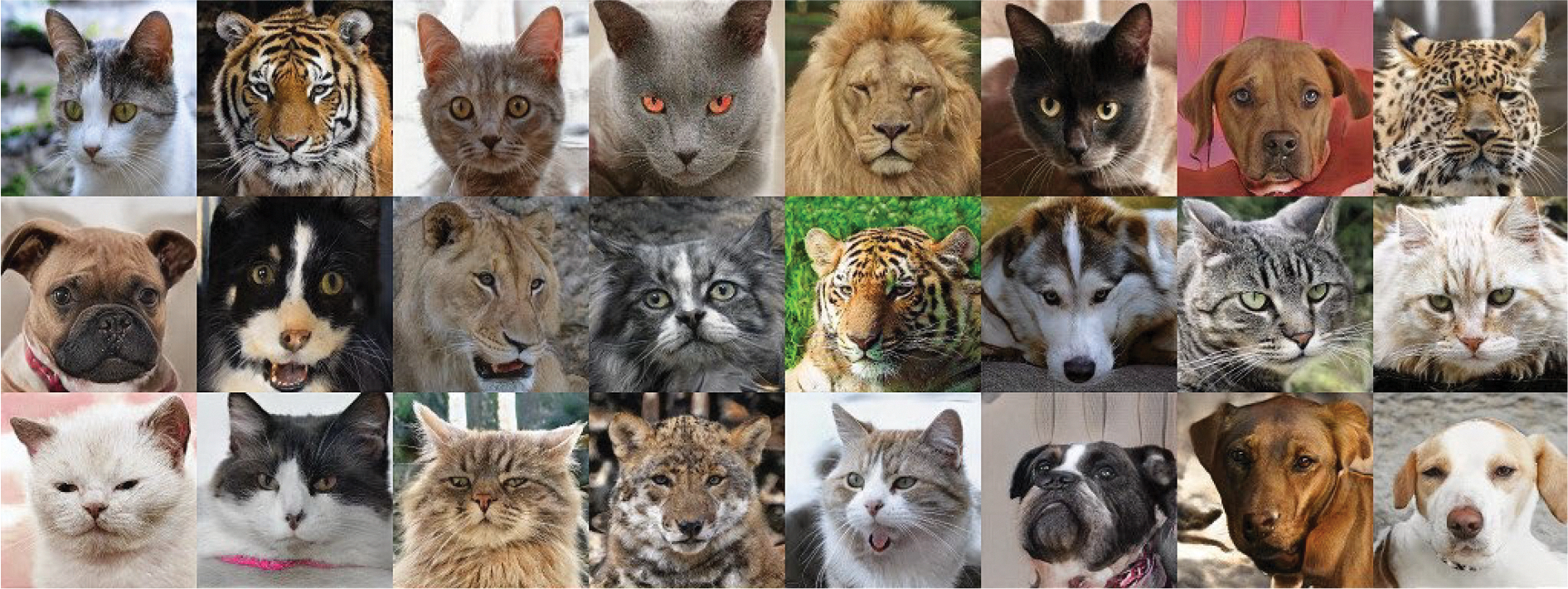
Animal-to-animal translation results with 8 × 8 × 128 intermediate features.

The experiment in this subsection is similar to the previous subsection. The only difference is the decrease in the intermediate feature from 16 × 16 × 128 to 8 × 8 × 128, which makes the encoder not able to reserve all features from the inputs. The result is shown in Fig. 11 and 12.

In face-to-face translation, the input image is in the first column, followed by the corresponding output images in the subsequent columns. Unlike in Fig. 9, the difference between each image in the same row is more pronounced. The image does not depict people with slightly different features. Instead, Fig. 11 shows people of different sex, gender, and other details. The common feature is that they take selfies from the same angle and maintain the same pose.

In Fig. 12, discerning the similarity becomes even more challenging. The first column shows the input, while the rest display the output. It shows that within the same row, the animal species and angles of the photos differ. However, we observed that, at the beginning of the training process, the network retains the pose and angle of the input image for animal data. However, as training progresses, these features are discarded to enhance the realism of the output image if the capacity of the network is insufficient. This is because the network is confused on which part of the feature should be preserved. This also shows that our analyses are correct.

## V. CONCLUSION

Our study provides new insights into the effectiveness of GANs in tasks involving image-to-image translation. We have shown that adversarial training, when applied to autoencoder models, can achieve results comparable to traditional methods without the necessity for additional complex loss penalties. Furthermore, we have explained the differences and similarities between GANs and autoencoders. We have also incorporated experimental results to demonstrate the validity of our findings.
